# Supplementary material for: Bothrops moojeni Venom and Its Components Strongly Affect Osteoclasts’ Maturation and Protein Patterns
Source: Toxins (Basel). 2021 Jun 30;13(7):459. doi: 10.3390/toxins13070459 (PMC8310197; doi:10.3390/toxins13070459)
Supplement: Supplementary file 1 [file toxins-13-00459-s001.zip › toxins-1254271-supplementary.pdf]

Supplementary Materials: *Bothrops moojeni* Venom and Its Components Strongly Affect Osteoclasts' Maturation and Protein Patterns

Fernanda D'Amélio, Hugo Vigerelli, Álvaro Rossan de Brandão Prieto-da-Silva, Eduardo Osório Frare, Isabel de Fátima Correia Batista, Daniel Carvalho Pimenta and Irina Kerkis

Table S1. Complete table of proteins identified by LC-MS.

Positive Control				
**Acession**	**Coverage (%)**	**Peptides**	**Avg Mass**	Description
P02768 I ALBU_HUMAN	63.21	3	69367	Albumin
P01023 I A2MG_HUMAN	34.89	1	163290	Alpha-2-macroglobulin
P20742 I PZP_HUMAN	34.89	1	163862	Pregnancy zone protein
P61026 I RAB10_HUMAN	29.24	1	22541	Ras-related protein Rab-10
Q86TE4 I LUZP2_HUMAN	29.24	1	38958	Leucine zipper protein 2
Q5VZM2 I RRAGB_HUMAN	29.24	1	43250	Ras-related GTP-binding protein B
Q13888 I TF2H2_HUMAN	29.24	1	44419	General transcription factor IIH subunit 2
O43323 I DHH_HUMAN	29.24	1	43577	Desert hedgehog protein
Q14623 I IHH_HUMAN	29.24	1	45251	Indian hedgehog protein
Q15465 I SHH_HUMAN	29.24	1	49607	Sonic hedgehog protein
Q8N118 I CP4X1_HUMAN	29.24	1	58875	Cytochrome P450 4X1
Q9NQV5 I PRD11_HUMAN	29.24	1	57863	PR domain-containing protein 11
P08582 I TRFM_HUMAN	29.24	1	80215	Melanotransferrin
O95025 I SEM3D_HUMAN	29.24	1	89651	Semaphorin-3D
Q12967 I GNDS_HUMAN	29.24	1	100607	Ral guanine nucleotide dissociation stimulator
Q9Y239 I NOD1_HUMAN	29.24	1	107691	Nucleotide-binding oligomerization domain-containing protein 1
Q15147 I PLCB4_HUMAN	29.24	1	134463	1-phosphatidylinositol 4,5-bisphosphate phosphodiesterase beta-4
Q92797 I SYMPK_HUMAN	29.24	1	141148	Symplekin
Q709C8 I VP13C_HUMAN	29.24	1	422393	Vacuolar protein sorting-associated protein 13C
O43422 I P52K_HUMAN	25.26	1	87704	52 kDa repressor of the inhibitor of the protein kinase
Q9BZ29 I DOCK9_HUMAN	25.26	1	236444	Dedicator of cytokinesis protein 9
O00370 I LORF2_HUMAN	23.93	1	149011	LINE-1 retrotransposable element ORF2 protein
Q96JQ0 I PCD16_HUMAN	23.93	1	346182	Protocadherin-16
Q9UPA5 I BSN_HUMAN	21.80	1	416475	Protein bassoon
A6ND36 I FA83G_HUMAN	21.09	1	90835	Protein FAM83G
Q9UKN7 I MYO15_HUMAN	39.19	3	395294	Unconventional myosin-XV
Q92817 I EVPL_HUMAN	25.17	1	231602	Envoplakin
Q9H098 I F107B_HUMAN	23.87	1	15558	Protein FAM107B
P50052 I AGTR2_HUMAN	23.87	1	41184	Type-2 angiotensin II receptor
Q02750 I MP2K1_HUMAN	23.87	1	43439	Dual specificity mitogen-activated protein kinase kinase 1

Q15438 | CYH1_HUMAN	23.87	1	46413	Cytohesin-1 OS=Homo sapiens OX=9606 GN=CYTH1 PE=1 SV=1
P38567 | HYALP_HUMAN	23.87	1	57848	Hyaluronidase PH-20
Q5TIA1 | MEI1_HUMAN	23.87	1	141161	Meiosis inhibitor protein 1
O00443 | P3C2A_HUMAN	23.87	1	190678	Phosphatidylinositol 4-phosphate 3-kinase C2 domain-containing subunit alpha
O00763 | ACACB_HUMAN	23.87	1	276539	Acetyl-CoA carboxylase 2
P29475 | NOS1_HUMAN	23.09	1	160970	Nitric oxide synthase, brain
Q8TD57 | DYH3_HUMAN	23.09	1	470774	Dynein heavy chain 3, axonemal
A7MD48 | SRRM4_HUMAN	23.01	1	68559	Serine/arginine repetitive matrix protein 4
Q58FF3 | ENPLL_HUMAN	22.81	1	45859	Putative endoplasmin-like protein
P14625 | ENPL_HUMAN	22.81	1	92469	Endoplasmin
Q96A65 | EXOC4_HUMAN	22.81	1	110498	Exocyst complex component 4
Q32MH5 | F214A_HUMAN	22.81	1	121670	Protein FAM214A
Q9Y618 | NCOR2_HUMAN	22.81	1	273654	Nuclear receptor corepressor 2
Q8N7J2 | AMER2_HUMAN	21.05	1	69507	APC membrane recruitment protein 2
Q8NEV8 | EXPH5_HUMAN	21.05	1	222517	Exophilin-5
Q16787 | LAMA3_HUMAN	20.43	1	366650	Laminin subunit alpha-3
Q14966 | ZN638_HUMAN	34.99	1	220623	Zinc finger protein 638
Q9BXT5 | TEX15_HUMAN	34.99	1	315336	Testis-expressed protein 15
Q96FA7 | ZB6CL_HUMAN	26.39	1	26352	ZBED6 C-terminal-like protein
O95072 | REC8_HUMAN	26.39	1	62614	Meiotic recombination protein REC8 homolog
Q6UX68 | XKR5_HUMAN	26.39	1	75014	XK-related protein 5
Q9HB58 | SP110_HUMAN	26.39	1	78396	Sp110 nuclear body protein
Q5VIR6 | VPS53_HUMAN	26.39	1	79653	Vacuolar protein sorting-associated protein 53 homolog
Q96K76 | UBP47_HUMAN	26.39	1	157311	Ubiquitin carboxyl-terminal hydrolase 47
O94813 | SLIT2_HUMAN	26.39	1	169869	Slit homolog 2 protein
Q5T4S7 | UBR4_HUMAN	26.39	1	573849	E3 ubiquitin-protein ligase UBR4
O75436 | VP26A_HUMAN	23.45	1	38170	Vacuolar protein sorting-associated protein 26A
Q2M3C7 | SPKAP_HUMAN	22.75	1	186455	A-kinase anchor protein SPHKAP
P52743 | ZN137_HUMAN	21.84	1	24115	Putative zinc finger protein 137
Q6AZW8 | ZN660_HUMAN	21.84	1	38270	Zinc finger protein 660
Q5T5D7 | ZN684_HUMAN	21.84	1	43945	Zinc finger protein 684
Q9ULI2 | RIMKB_HUMAN	21.84	1	42464	Beta-citrylglutamate synthase B
O76081 | RGS20_HUMAN	21.84	1	43692	Regulator of G-protein signaling 20
B4DX44 | ZN736_HUMAN	21.84	1	49868	Zinc finger protein 736
Q9UC06 | ZNF70_HUMAN	21.84	1	50802	Zinc finger protein 70
O95219 | SNX4_HUMAN	21.84	1	51909	Sorting nexin-4
Q9Y5X2 | SNX8_HUMAN	21.84	1	52569	Sorting nexin-8
Q03924 | ZN117_HUMAN	21.84	1	56376	Zinc finger protein 117
P49910 | ZN165_HUMAN	21.84	1	55771	Zinc finger protein 165
Q96NJ6 | ZFP3_HUMAN	21.84	1	57662	Zinc finger protein 3 homolog
A2RRD8 | ZN320_HUMAN	21.84	1	59326	Zinc finger protein 320
Q7Z3I7 | ZN572_HUMAN	21.84	1	61238	Zinc finger protein 572
Q9BRR0 | ZKSC3_HUMAN	21.84	1	60641	Zinc finger protein with KRAB and SCAN domains 3

Q969J2 l ZKSC4_HUMAN	21.84	1	61579	Zinc finger protein with KRAB and SCAN domains 4
Q5TEC3 l ZN697_HUMAN	21.84	1	60461	Zinc finger protein 697
P17098 l ZNF8_HUMAN	21.84	1	64970	Zinc finger protein 8
Q8N184 l ZN567_HUMAN	21.84	1	75164	Zinc finger protein 567
Q96GC6 l ZN274_HUMAN	21.84	1	74177	Neurotrophin receptor-interacting factor homolog
P17021 l ZNF17_HUMAN	21.84	1	77204	Zinc finger protein 17
P52740 l ZN132_HUMAN	21.84	1	80623	Zinc finger protein 132
Q96SZ4 l ZSC10_HUMAN	21.84	1	80387	Zinc finger and SCAN domain-containing protein 10
P78563 l RED1_HUMAN	21.84	1	80763	Double-stranded RNA-specific editase 1
Q14586 l ZN267_HUMAN	21.84	1	87376	Zinc finger protein 267
Q9ULD5 l ZN777_HUMAN	21.84	1	85176	Zinc finger protein 777
P57078 l RIPK4_HUMAN	21.84	1	91611	Receptor-interacting serine/threonine-protein kinase 4
Q8NDZ2 l SIMC1_HUMAN	21.84	1	96838	SUMO-interacting motif-containing protein 1
O14827 l RGRF2_HUMAN	21.84	1	140764	Ras-specific guanine nucleotide-releasing factor 2
Q13972 l RGRF1_HUMAN	21.84	1	145233	Ras-specific guanine nucleotide-releasing factor 1
O43149 l ZZEF1_HUMAN	21.84	1	331075	Zinc finger ZZ-type and EF-hand domain-containing protein 1
Q9NYU1 l UGGG2_HUMAN	21.84	1	174734	UDP-glucose:glycoprotein glucosyltransferase 2
Q9HCD6 l TANC2_HUMAN	21.84	1	219647	Protein TANC2
Q6ZQQ6 l WDR87_HUMAN	21.84	1	333185	WD repeat-containing protein 87
Q63HK3 l ZKSC2_HUMAN	21.77	1	110941	Zinc finger protein with KRAB and SCAN domains 2
Q7Z2W7 l TRPM8_HUMAN	21.77	1	127685	Transient receptor potential cation channel subfamily M member 8
O95359 l TACC2_HUMAN	21.53	1	309426	Transforming acidic coiled-coil-containing protein 2
A6NJY1 l SL9P1_HUMAN	26.55	1	30828	Putative SLC9B1-like protein SLC9B1P1
Q96EF9 l ZHX1R_HUMAN	26.55	1	33285	Zinc fingers and homeoboxes protein 1, isoform 2
Q96TC7 l RMD3_HUMAN	26.55	1	52118	Regulator of microtubule dynamics protein 3
P98170 l XIAP_HUMAN	26.55	1	56685	E3 ubiquitin-protein ligase XIAP
Q4ZJI4 l SL9B1_HUMAN	26.55	1	56054	Sodium/hydrogen exchanger 9B1
P26639 l SYTC_HUMAN	26.55	1	83435	Threonine--tRNA ligase, cytoplasmic
Q5T1R4 l ZEP3_HUMAN	26.55	1	259462	Transcription factor HIVEP3
P31629 l ZEP2_HUMAN	26.55	1	269051	Transcription factor HIVEP2
P15822 l ZEP1_HUMAN	26.55	1	296863	Zinc finger protein 40
P22570 l ADRO_HUMAN	22.62	1	53837	NADPH:adrenodoxin oxidoreductase, mitochondrial
Q08188 l TGM3_HUMAN	22.62	1	76632	Protein-glutamine gamma-glutamyltransferase E
O95382 l M3K6_HUMAN	22.62	1	142596	Mitogen-activated protein kinase kinase kinase 6

Q8NGJ9	O51T1_HUMAN	21.70	1	36998	Olfactory receptor 51T1
P49221	TGM4_HUMAN	21.70	1	77145	Protein-glutamine gamma-glutamyltransferase 4
B5MCY1	TDR15_HUMAN	21.70	1	221720	Tudor domain-containing protein 15
P13489	RINI_HUMAN	21.42	1	49974	Ribonuclease inhibitor
B4DYI2	S31C2_HUMAN	21.42	1	124429	Putative spermatogenesis-associated protein 31C2
P0DKV0	S31C1_HUMAN	21.42	1	130522	Putative spermatogenesis-associated protein 31C1
Q5VVP1	S31A6_HUMAN	21.42	1	147817	Spermatogenesis-associated protein 31A6
Q5VYP0	S31A3_HUMAN	21.42	1	148706	Spermatogenesis-associated protein 31A3
Q8IWB4	S31A7_HUMAN	21.42	1	148734	Spermatogenesis-associated protein 31A7
Q5TZJ5	S31A1_HUMAN	21.42	1	148560	Spermatogenesis-associated protein 31A1
Q5VU36	S31A5_HUMAN	21.42	1	148686	Spermatogenesis-associated protein 31A5
Q7Z2Z1	TICRR_HUMAN	21.35	1	210855	Treslin
Q9C0G6	DYH6_HUMAN	21.35	1	475986	Dynein heavy chain 6, axonemal

Negative Control

Acession	Coverage (%)	Peptides	Avg Mass	Description
Q9UKN7	MYO15_HUMAN	39.19	1	395294	Unconventional myosin-XV
Q92817	EVPL_HUMAN	25.17	0	231602	Envoplakin
Q9H098	F107B_HUMAN	23.87	4	15558	Protein FAM107B
P50052	AGTR2_HUMAN	23.87	1	41184	Type-2 angiotensin II receptor
Q02750	MP2K1_HUMAN	23.87	1	43439	Dual specificity mitogen-activated protein kinase kinase 1
Q15438	CYH1_HUMAN	23.87	1	46413	Cytohesin-1
P38567	HYALP_HUMAN	23.87	1	57848	Hyaluronidase PH-20
Q5TIA1	MEI1_HUMAN	23.87	0	141161	Meiosis inhibitor protein 1
O00443	P3C2A_HUMAN	23.87	0	190678	Phosphatidylinositol 4-phosphate 3-kinase C2 domain-containing subunit alpha
O00763	ACACB_HUMAN	23.87	0	276539	Acetyl-CoA carboxylase 2
P29475	NOS1_HUMAN	23.09	0	160970	Nitric oxide synthase, brain
Q8TD57	DYH3_HUMAN	23.09	0	470774	Dynein heavy chain 3, axonemal
A7MD48	SRRM4_HUMAN	23.01	1	68559	Serine/arginine repetitive matrix protein 4
Q58FF3	ENPLL_HUMAN	22.81	1	45859	Putative endoplasmin-like protein
P14625	ENPL_HUMAN	22.81	1	92469	Endoplasmin
Q96A65	EXOC4_HUMAN	22.81	1	110498	Exocyst complex component 4
Q32MH5	F214A_HUMAN	22.81	0	121670	Protein FAM214A
Q9Y618	NCOR2_HUMAN	22.81	0	273654	Nuclear receptor corepressor 2
Q8N7J2	AMER2_HUMAN	21.05	1	69507	APC membrane recruitment protein 2
Q8NEV8	EXPH5_HUMAN	21.05	0	222517	Exophilin-5
Q16787	LAMA3_HUMAN	20.43	0	366650	Laminin subunit alpha-3

A5PLK6|RGSL_HUMAN	24.91	0	125688	Regulator of G-protein signaling protein-like
Q86YW5|TRML1_HUMAN	24.91	2	32679	Trem-like transcript 1 protein
P29622|KAIN_HUMAN	24.91	1	48542	Kallistatin
Q9BQC3|DPH2_HUMAN	24.91	1	52083	2-(3-amino-3-carboxypropyl)histidine synthase subunit 2
Q6ZNC8|MBOA1_HUMAN	24.91	1	56557	Lysophospholipid acyltransferase 1
Q6ZUJ8|BCAP_HUMAN	24.91	1	90398	Phosphoinositide 3-kinase adapter protein 1
O00206|TLR4_HUMAN	24.91	1	95680	Toll-like receptor 4
Q96PV7|F193B_HUMAN	24.91	1	96543	Protein FAM193B
Q9BZ76|CNTP3_HUMAN	24.91	0	140690	Contactin-associated protein-like 3
Q96NU0|CNT3B_HUMAN	24.91	0	140414	Contactin-associated protein-like 3B
Q9ULH0|KDIS_HUMAN	24.91	0	196541	Kinase D-interacting substrate of 220 kDa
Q9HCM3|K1549_HUMAN	24.91	0	210753	UPF0606 protein KIAA1549
Q8IVV2|LOXH1_HUMAN	24.91	0	235676	Lipoxygenase homology domain-containing protein 1
Q5T5X7|BEND3_HUMAN	21.23	1	94475	BEN domain-containing protein 3
Q8IWI9|MGAP_HUMAN	30.09	0	336160	MAX gene-associated protein
Q8NG48|LINES_HUMAN	24.28	1	85857	Protein Lines homolog 1
Q86UT6|NLRX1_HUMAN	24.28	1	107616	NLR family member X1
P24311|COX7B_HUMAN	23.01	6	9161	Cytochrome c oxidase subunit 7B, mitochondrial
Q8TF08|CX7B2_HUMAN	23.01	6	9077	Cytochrome c oxidase subunit 7B2, mitochondrial
Q9NXE4|NSMA3_HUMAN	23.01	1	97810	Sphingomyelin phosphodiesterase 4
Q8WUM0|NU133_HUMAN	23.01	0	128979	Nuclear pore complex protein Nup133
Q504Q3|PAN2_HUMAN	23.01	0	135367	PAN2-PAN3 deadenylation complex catalytic subunit PAN2
Q6ZU69|F205A_HUMAN	23.01	0	148095	Protein FAM205A
Q9UKK3|PARP4_HUMAN	21.85	0	192593	Protein mono-ADP-ribosyltransferase PARP4
Q9GZV4|IF5A2_HUMAN	21.58	3	16793	Eukaryotic translation initiation factor 5A-2
P63241|IF5A1_HUMAN	21.58	3	16832	Eukaryotic translation initiation factor 5A-1
Q6IS14|IF5AL_HUMAN	21.58	3	16773	Eukaryotic translation initiation factor 5A-1-like
P56749|CLD12_HUMAN	21.58	2	27110	Claudin-12
Q66K74|MAP1S_HUMAN	21.58	0	112211	Microtubule-associated protein 1S
Q9NS87|KIF15_HUMAN	20.50	0	160159	Kinesin-like protein KIF15
Q96PG2|M4A10_HUMAN	20.10	2	29747	Membrane-spanning 4-domains subfamily A member 10
O43422|P52K_HUMAN	20.10	1	87704	52 kDa repressor of the inhibitor of the protein kinase

Q15811ǀITSN1_HUMAN	20.10	0	195420	Intersectin-1
Q9Y4F3ǀMARF1_HUMAN	20.10	0	192858	Meiosis regulator and mRNA stability factor 1
Q92608ǀDOCK2_HUMAN	20.10	0	211947	Dedicator of cytokinesis protein 2
Bothrops moojeni				
Acession	Coverage (%)	Peptides	Avg Mass	Description
P02768ǀALBU_HUMAN	72.38	6	69367	Albumin
P02787ǀTRFE_HUMAN	37.29	1	77064	Serotransferrin
Q9UBC5ǀMYO1A_HUMAN	33.17	1	118401	Unconventional myosin-Ia
Q8N682ǀDRAM1_HUMAN	26.38	2	26253	DNA damage-regulated autophagy modulator protein 1
Q8N6G2ǀTEX26_HUMAN	26.38	2	33595	Testis-expressed protein 26
Q5T6L9ǀEMARD_HUMAN	26.38	1	77788	Endoplasmic reticulum membrane-associated RNA degradation protein
P17948ǀVGFR1_HUMAN	26.38	0	150768	Vascular endothelial growth factor receptor 1
Q9P219ǀDAPLE_HUMAN	26.38	0	228228	Protein Daple
A2RRP1ǀNBAS_HUMAN	26.38	0	268569	Neuroblastoma-amplified sequence
Q6ZP29ǀLAAT1_HUMAN	25.74	2	31947	Lysosomal amino acid transporter 1 homolog
Q96I82ǀKAZD1_HUMAN	25.74	2	32945	Kazal-type serine protease inhibitor domain-containing protein 1
Q99645ǀEPYC_HUMAN	25.74	2	36637	Epiphycan
Q9HBL6ǀLRTM1_HUMAN	25.74	1	38171	Leucine-rich repeat and transmembrane domain-containing protein 1
Q8N3X6ǀLCORL_HUMAN	25.74	1	66964	Ligand-dependent nuclear receptor corepressor-like protein
Q12882ǀDPYD_HUMAN	25.74	0	111401	Dihydropyrimidine dehydrogenase [NADP(+)]
Q29RF7ǀPDS5A_HUMAN	25.74	0	150830	Sister chromatid cohesion protein PDS5 homolog A
O94813ǀSLIT2_HUMAN	25.74	0	169869	Slit homolog 2 protein
A6NKB5ǀPCX2_HUMAN	25.74	0	237275	Pecanex-like protein 2
Q92610ǀZN592_HUMAN	21.42	0	137528	Zinc finger protein 592
Q9UIY3ǀRWD2A_HUMAN	38.26	3	33893	RWD domain-containing protein 2A
Q8NHG8ǀZNRF2_HUMAN	29.86	4	24115	E3 ubiquitin-protein ligase ZNRF2
Q99996ǀAKAP9_HUMAN	29.73	0	452990	A-kinase anchor protein 9
P00748ǀFA12_HUMAN	27.17	1	67792	Coagulation factor XII
Q8NEP7ǀKLDC9_HUMAN	24.68	1	37754	Kelch domain-containing protein 9
Q6UY14ǀATL4_HUMAN	24.68	0	116545	ADAMTS-like protein 4
Q92608ǀDOCK2_HUMAN	24.19	0	211947	Dedicator of cytokinesis protein 2
Q5XG92ǀEST4A_HUMAN	23.30	1	63529	Carboxylesterase 4A
Q8N4P2ǀTT30B_HUMAN	23.30	1	76099	Tetratricopeptide repeat protein 30B

Q86WT1∣TT30A_HUMAN	23.30	1	76136	Tetratricopeptide repeat protein 30A
Q6ZP29∣LAAT1_HUMAN	22.64	2	31947	Lysosomal amino acid transporter 1 homolog
Q99645∣EPYC_HUMAN	22.64	2	36637	Epiphycan
Q8N3X6∣LCORL_HUMAN	22.64	1	66964	Ligand-dependent nuclear receptor corepressor-like protein
Q9C099∣LRCC1_HUMAN	22.64	0	119596	OS=Homo sapiens OX=9606 GN=LRRCC1 PE=1 SV=2
Q96RV3∣PCX1_HUMAN	22.64	0	258673	Pecanex-like protein 1
Q5T4S7∣UBR4_HUMAN	22.64	0	573849	E3 ubiquitin-protein ligase UBR4
Q9BZE4∣NOG1_HUMAN	22.24	1	73965	Nucleolar GTP-binding protein 1
Q8IYB7∣DI3L2_HUMAN	22.24	1	99279	DIS3-like exonuclease 2
Q9BRR8∣GPTC1_HUMAN	21.89	1	103345	G patch domain-containing protein 1
Q8IWZ8∣SUGP1_HUMAN	21.89	1	72471	SURP and G-patch domain-containing protein 1
Q8N302∣AGGF1_HUMAN	21.89	1	80977	Angiogenic factor with G patch and FHA domains 1
Q8IX01∣SUGP2_HUMAN	21.89	0	120207	SURP and G-patch domain-containing protein 2
O75369∣FLNB_HUMAN	21.85	0	278162	Filamin-B
Q8WZ55∣BSND_HUMAN	20.21	2	35197	Barttin
O94854∣K0754_HUMAN	20.21	0	135148	Uncharacterized protein KIAA0754
Q14008∣CKAP5_HUMAN	20.21	0	225493	Cytoskeleton-associated protein 5
Q86VR8∣FJX1_HUMAN	20.16	1	48507	Four-jointed box protein 1
O60437∣PEPL_HUMAN	33.84	1	204745	Periplakin
Q13224∣NMDE2_HUMAN	29.62	1	166367	Glutamate receptor ionotropic, NMDA 2B
Q8NHG8∣ZNRF2_HUMAN	27.36	4	24115	E3 ubiquitin-protein ligase ZNRF2
P62805∣H4_HUMAN	25.96	5	11367	Histone H4
Q9Y320∣TMX2_HUMAN	25.96	2	34038	Thioredoxin-related transmembrane protein 2
P20042∣IF2B_HUMAN	25.96	2	38388	Eukaryotic translation initiation factor 2 subunit 2
Q6ZSC3∣RBM43_HUMAN	25.96	1	40666	RNA-binding protein 43
Q96FI4∣NEIL1_HUMAN	25.96	1	43684	Endonuclease 8-like 1
Q9Y4A0∣JERKL_HUMAN	25.96	1	59912	Jerky protein homolog-like
Q9H078∣CLPB_HUMAN	25.96	1	78729	Caseinolytic peptidase B protein homolog
Q9UK96∣FBX10_HUMAN	25.96	1	105195	F-box only protein 10
Q9UJF2∣NGAP_HUMAN	25.96	0	128557	Ras GTPase-activating protein nGAP
Q9HCH0∣NCK5L_HUMAN	25.96	0	139013	Nck-associated protein 5-like
Q13395∣TARB1_HUMAN	25.96	0	181674	Probable methyltransferase TARBP1
Q8NDH2∣CC168_HUMAN	25.96	0	277949	Coiled-coil domain-containing protein 168
H3BQW9∣F229A_HUMAN	22.95	4	12972	Protein FAM229A
Q96LR7∣CB050_HUMAN	22.95	3	17838	Uncharacterized protein C2orf50
P22674∣CCNO_HUMAN	22.95	1	38096	Cyclin-O

Q567U6 | CCD93_HUMAN	22.95	1	73198	Coiled-coil domain-containing protein 93
Q12894 | IFRD2_HUMAN	22.95	1	54814	Interferon-related developmental regulator 2
O14511 | NRG2_HUMAN	22.95	1	91679	Pro-neuregulin-2, membrane-bound isoform
O95714 | HERC2_HUMAN	22.95	0	527234	E3 ubiquitin-protein ligase HERC2
Q9BXM0 | PRAX_HUMAN	22.95	0	154904	Periaxin
Q9Y4B5 | MTCL1_HUMAN	22.95	0	209525	Microtubule cross-linking factor 1
Q9P0X4 | CAC1I_HUMAN	22.95	0	245101	Voltage-dependent T-type calcium channel subunit alpha-1I
P43652 | AFAM_HUMAN	22.81	1	69069	Afamin
P0C221 | CC175_HUMAN	22.72	1	93626	Coiled-coil domain-containing protein 175
Q3KNW1 | SNAI3_HUMAN	22.48	2	32474	Zinc finger protein SNAI3
P51161 | FABP6_HUMAN	22.23	4	14371	Gastrotropin
A6NC98 | CC88B_HUMAN	22.23	0	164808	Coiled-coil domain-containing protein 88B
Q8IXQ8 | PDZD9_HUMAN	22.04	2	29904	PDZ domain-containing protein 9
Q92599 | SEPT8_HUMAN	22.04	1	55756	Septin-8
P27816 | MAP4_HUMAN	22.04	0	121005	Microtubule-associated protein 4
Q05C16 | LRC63_HUMAN	21.92	1	66309	Leucine-rich repeat-containing protein 63
Q86VS8 | HOOK3_HUMAN	21.92	1	83126	Protein Hook homolog 3
Q6NUT2 | D19L2_HUMAN	21.92	1	87374	Probable C-mannosyltransferase DPY19L2
Q14204 | DYHC1_HUMAN	21.92	0	532412	Cytoplasmic dynein 1 heavy chain 1
A4UGR9 | XIRP2_HUMAN	21.92	0	382302	Xin actin-binding repeat-containing protein 2
Q07890 | SOS2_HUMAN	21.92	0	152978	Son of sevenless homolog 2
Q6ZR08 | DYH12_HUMAN	21.92	0	356942	Dynein heavy chain 12, axonemal
Q460N5 | PAR14_HUMAN	21.92	0	202799	Protein mono-ADP-ribosyltransferase PARP14
O15078 | CE290_HUMAN	21.92	0	290384	Centrosomal protein of 290 kDa
Q99996 | AKAP9_HUMAN	21.92	0	452990	A-kinase anchor protein 9
Q9UPU3 | SORC3_HUMAN	21.42	1	135755	VPS10 domain-containing receptor SorCS3
O95150 | TNF15_HUMAN	21.32	3	28087	Tumor necrosis factor ligand superfamily member 15
P57057 | G6PT2_HUMAN	20.54	1	57648	Glucose-6-phosphate exchanger SLC37A1
Q9H4Q3 | PRD13_HUMAN	20.54	1	73981	PR domain zinc finger protein 13
Q96AQ6 | PBIP1_HUMAN	20.18	1	80643	Pre-B-cell leukemia transcription factor-interacting protein 1
P02765 | FETUA_HUMAN	39.47	5	39325	Alpha-2-HS-glycoprotein
Q7Z5K2 | WAPL_HUMAN	20.55	1	132945	Wings apart-like protein homolog

Q86WS5丨TMPSC_HUMAN	20.15	2	38605	Transmembrane protease serine 12

High mass *Bothrops moojeni*

Acession	Coverage (%)	Peptides	Avg mass	Description
P24390丨ERD21_HUMAN	36.71	2	24542	ER lumen protein-retaining receptor 1
Q7Z412丨PEX26_HUMAN	36.71	2	33898	Peroxisome assembly protein 26
Q969N4丨TAAR8_HUMAN	36.71	1	38029	Trace amine-associated receptor 8
P51686丨CCR9_HUMAN	36.71	1	42016	C-C chemokine receptor type 9
Q96P65丨QRFPR_HUMAN	36.71	1	49488	Pyroglutamylated RF-amide peptide receptor
Q2WGJ6丨KLH38_HUMAN	36.71	1	65541	Kelch-like protein 38
Q8WYR1丨PI3R5_HUMAN	36.71	1	97348	Phosphoinositide 3-kinase regulatory subunit 5
O15228丨GNPAT_HUMAN	36.71	1	77188	Dihydroxyacetone phosphate acyltransferase
P53675丨CLH2_HUMAN	36.71	0	187029	Clathrin heavy chain 2
Q14997丨PSME4_HUMAN	36.71	0	211332	Proteasome activator complex subunit 4
Q6ZP29丨LAAT1_HUMAN	28.15	2	31947	Lysosomal amino acid transporter 1 homolog
Q96I82丨KAZD1_HUMAN	28.15	2	32945	Kazal-type serine protease inhibitor domain-containing protein 1
Q99645丨EPYC_HUMAN	28.15	2	36637	Epiphycan
Q9HBL6丨LRTM1_HUMAN	28.15	1	38171	Leucine-rich repeat and transmembrane domain-containing protein 1
Q8N3X6丨LCORL_HUMAN	28.15	1	66964	Ligand-dependent nuclear receptor corepressor-like protein
Q12882丨DPYD_HUMAN	28.15	0	111401	Dihydropyrimidine dehydrogenase [NADP(+)]
Q29RF7丨PDS5A_HUMAN	28.15	0	150830	Sister chromatid cohesion protein PDS5 homolog A
O94813丨SLIT2_HUMAN	28.15	0	169869	Slit homolog 2 protein
A6NKB5丨PCX2_HUMAN	28.15	0	237275	Pecanex-like protein 2
Q9H6B4丨CLMP_HUMAN	21.80	2	41281	CXADR-like membrane protein
Q9UPA5丨BSN_HUMAN	20.09	0	416475	Protein bassoon

Low Mass *Bothrops moojeni*

Acession	Coverage (%)	Peptides	Avg Mass	Description
P01023丨A2MG_HUMAN	40.46	1	163290	Alpha-2-macroglobulin
P20742丨PZP_HUMAN	40.46	1	163862	Pregnancy zone protein
P26436丨ASPX_HUMAN	29.37	2	28156	Acrosomal protein SP-10
P52306丨GDS1_HUMAN	29.37	1	66317	Rap1 GTPase-GDP dissociation stimulator 1
P35555丨FBN1_HUMAN	29.37	0	312297	Fibrillin-1
P61026丨RAB10_HUMAN	29.34	2	22541	Ras-related protein Rab-10
Q13888丨TF2H2_HUMAN	29.34	1	44419	General transcription factor IIH subunit 2
O43323丨DHH_HUMAN	29.34	1	43577	Desert hedgehog protein
P02787丨TRFE_HUMAN	29.34	1	77064	Serotransferrin

Q9Y239 I NOD1_HUMAN	29.34	1	107691	Nucleotide-binding oligomerization domain-containing protein 1
Q92797 I SYMPK_HUMAN	29.34	0	141148	Symplekin
Q7Z460 I CLAP1_HUMAN	27.82	0	169450	CLIP-associating protein 1
O43422 I P52K_HUMAN	25.30	1	87704	52 kDa repressor of the inhibitor of the protein kinase
Q9BZ29 I DOCK9_HUMAN	25.30	0	236444	Dedicator of cytokinesis protein 9
Q8WVD5 I RN141_HUMAN	25.07	2	25535	RING finger protein 141
Q9H2P9 I DPH5_HUMAN	25.07	2	31651	Diphthine methyl ester synthase
Q9NU02 I ANKE1_HUMAN	25.07	1	86664	Ankyrin repeat and EF-hand domain-containing protein 1
Q8TF40 I FNIP1_HUMAN	25.07	0	130555	Folliculin-interacting protein 1
Q6ZUT9 I DEN5B_HUMAN	25.07	0	145019	DENN domain-containing protein 5B
Q86XX4 I FRAS1_HUMAN	25.07	0	443217	Extracellular matrix protein FRAS1
Q8NEZ4 I KMT2C_HUMAN	20.35	0	541376	Histone-lysine N-methyltransferase 2C
